# Supplementary material for: Expression of CD226 is associated to but not required for NK cell education
Source: Nat Commun. 2017 May 31;8:15627. doi: 10.1038/ncomms15627 (PMC5460037; doi:10.1038/ncomms15627)
Supplement: Supplementary Information — Supplementary Figures, Supplementary Note and Supplementary Reference. [file ncomms15627-s1.pdf]

Supplementary Material

Expression of DNAM-1 (CD226) is associated to but not required for NK cell education

Multivariate Analysis

To further examine the effect on DNAM-1 imposed by education, we repeated the multivariate analysis (PCA, Fig. 4) using a different clustering algorithm, OPLSDA (orthogonal projection and latent structures discriminant analysis). This is an algorithm optimized for finding differences between pre-defined groups, which takes the mouse strain (class membership) into account and can thereby provide more information on inter-group variation[1]. The PCA in Fig. 4 was performed using the proportion of DNAM-1^+ cells of every of the 32 NK cell subset based on expression of Ly49r and NKG2A (for gating see Suppl. Fig. 2a). Every dot represents an individual mouse. Assignment to mouse strains was not part of the calculation and has been solely used for color-coding. For the OPLSDA (Suppl. Fig. 5b-c), group assignment was used for calculating intergroup variation and subsequent clustering. In an OPLS-DA, a test set (a randomly chosen part of the entire dataset) is used to estimate the predictability of another set (a different randomly chosen part of the dataset). This is done in an iterative way, so that every possible division of the entire dataset is used for the estimate of predictability.

We excluded all subsets positive for NKG2A, as the clustering of the mouse strains seems to be mainly driven by the expression of Ly49r, and since we propose that the maturation-dependent down-regulation of DNAM-1 (which is mainly seen in the NKG2A^+ NK cell subsets) obscures the education-dependent effect on DNAM-1 expression. Furthermore, we analyzed additional mouse strains displaying expression of multiple MHC-I alleles. By focusing on the Ly49r^+NKG2A^- NK subsets, and using a supervised clustering method, we got a clearer picture with more distinct clusters (Suppl. Fig. 4b). This led to a slightly reduced range of variation (compare scale in Suppl. Fig. 4b and Fig. 4a); in particular in the score t1 direction (the scores t1 and t2 are the new variables generated by dimension-reduction in OPLS-DA), but also in t2 (education via Ly49A/G versus Ly49C/I).

Interestingly, in the OPLS-DA the location of the $K^{b-/-}$ mice moves towards negative values in t2

direction, which means that in the absence of NKG2A, the potential binding of Ly49rs to D^b becomes apparent. This was visualized by plotting the coefficients that contribute to t2 for every individual mouse strain (Fig. 4c). The size of the coefficient represents the change in the Y-variable when the X-variable varies. Thus, these coefficients express how strongly Y is correlated to the X-variables. The error bars indicate the confidence intervals of the coefficients. The coefficient is significant when the error bar does not include zero (above the noise). The coefficients have been sorted in a descending manner, so that coefficients with large contribution are found to the left of each plot.

Note that B6 and $D^{b-/-}$ mice look very similar, and MHC-I-deficient D^d-transgenic mice ($K^{b-/-}D^{b-/-}D^{d+/+}$), D^d-transgenic ($K^{b+/+}D^{b+/+}D^{d+/+}$) and $K^{b-/-}$ mice look alike. For $K^{b-/-}$ mice the contribution of the Ly49A, Ly49G, Ly49A/G subsets is less than for MHC-I-deficient D^d-transgenic mice ($K^{b-/-}D^{b-/-}D^{d+/+}$) (as the binding to D^b is weaker than to D^d) and for D^d-transgenic ($K^{b+/+}D^{b+/+}D^{d+/+}$) mice. As expected, the placement of the $D^{b-/-}$ and B6 clusters in t2 is mainly driven by DNAM-1 expression on Ly49C and Ly49I positive subsets, and their lack of DNAM-1 on Ly49A and Ly49G positive subsets (compared to the other strains). For mice expressing the MHCI allele D^d, it is vice versa, while for $K^{b-/-}D^{b-/-}$ mice, t2 contributes negatively (less DNAM-1 on all subsets compared to the other strains). To our surprise, for $K^{b-/-}$ mice, not only the Ly49A-positive subsets, but also the Ly49G-positive subset significant characterizes this mouse strain in relation to others (error bar does not reach 0). This could imply a possible binding of Ly49G2 to D^b.

Reference

1 Worley, B. & Powers, R. Multivariate Analysis in Metabolomics. *Current Metabolomics* **1**, 92-107, doi:10.2174/2213235X11301010092 (2013).

Supplementary Figures

Supplementary Figure 1

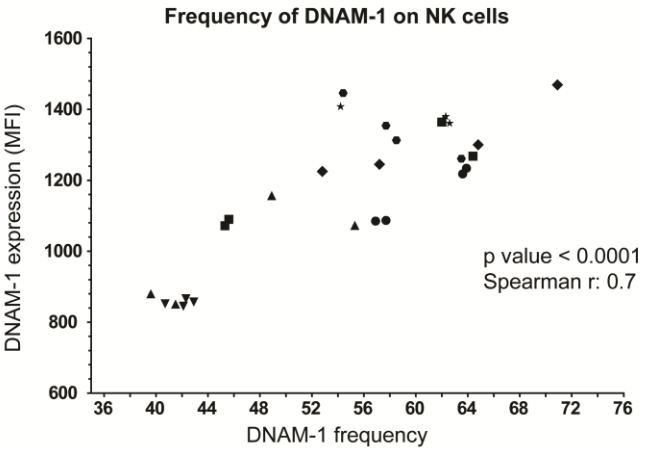

Supplementary Figure 1. Proportion of of DNAM-1^{+} cells and expression level per cell (MFI) correlate. NK cells of $B2m^{-/-}$ (triangles, down), $K^{b-/-}D^{b-/-}$ (triangles, up), $K^{b-/-}$ (squares), $D^{b-/-}$ (diamonds), B6 (circles), MHC-I-deficient D^{d}-transgenic mice ($K^{b-/-}D^{b-/-}D^{d+/+}$) (hexagons) and D^{d}-transgenic ($K^{b+/+}D^{b+/+}D^{d+/+}$) mice (stars) were stained for DNAM-1 and the proportion of DNAM-1^{+} cells was correlated to the MFI of DNAM-1^{+} NK cells. Results shown are from one representative experiment with 4 mice per group. Significant correlation was calculated by spearman test, r= 0.7 and a p-value ***p<0.001.

Supplementary Figure 2

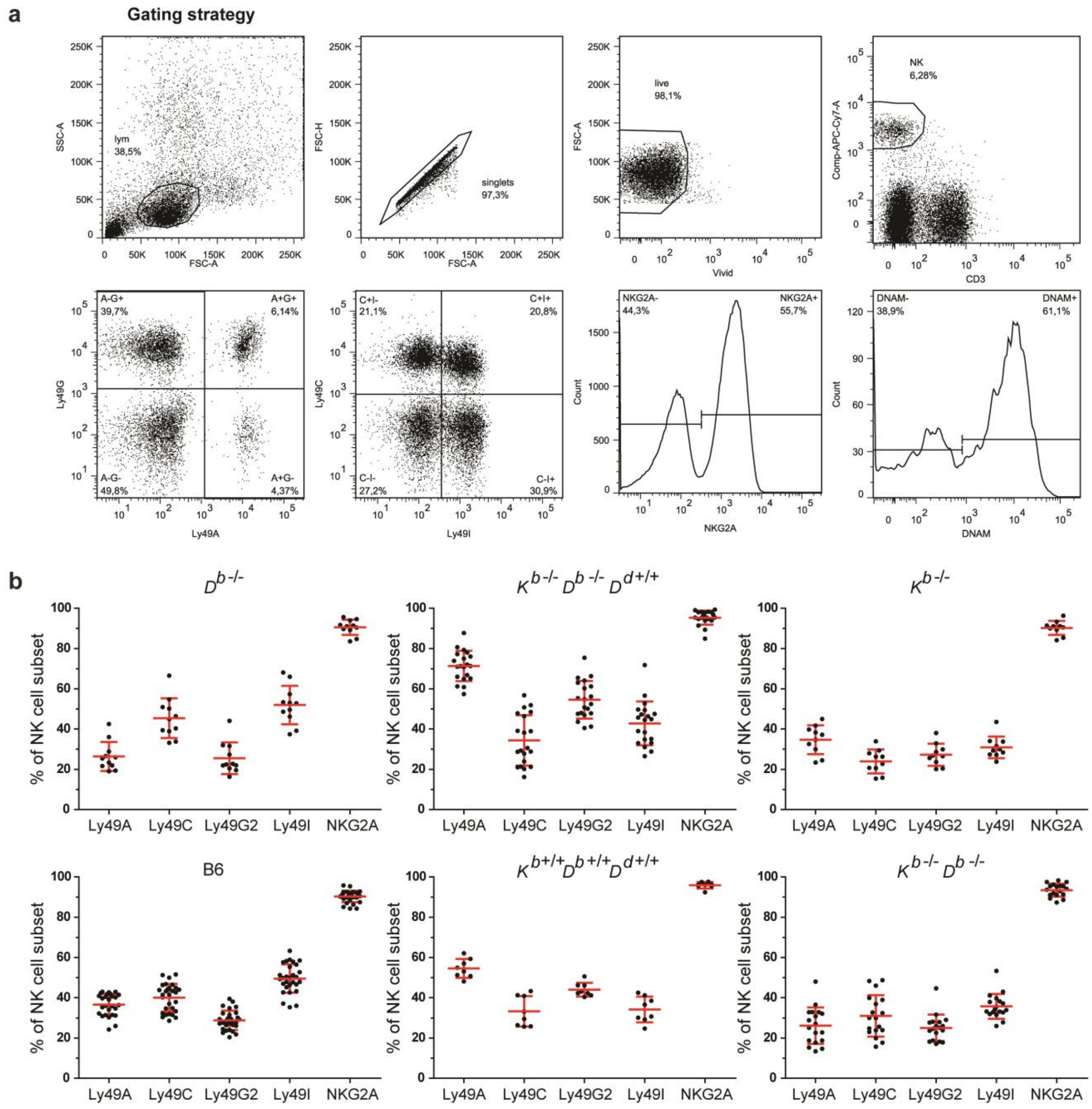

Supplementary Figure 2. Expression of DNAM-1 and inhibitory receptors on NK cells. (a) Gating strategy for Fig. 2. Cells are gated on lymphocytes by forward and side scatter properties, then on single cells, live cells as determined by live/dead cell staining. NK cells are defined as $CD3^-NK1.1^+$ and gated successively based on expression of inhibitory receptors Ly49A, Ly49G2, Ly49C, Ly49I and NKG2A and then DNAM-1. **(b)** Frequency of DNAM-1^+ NK cell subsets based on expression on one single inhibitory receptor in $D^{b-/-}$, MHC-I-deficient

D^d-transgenic mice ($K^{b-/-}D^{b-/-}D^{d+/+}$), $K^{b-/-}$, B6 (K^bD^b), D^d-transgenic ($K^{b+/+}D^{b+/+}D^{d+/+}$) and $K^{b-/-}D^{b-/-}$ mice. Data are from 3 experiments with at least 6 mice in total.

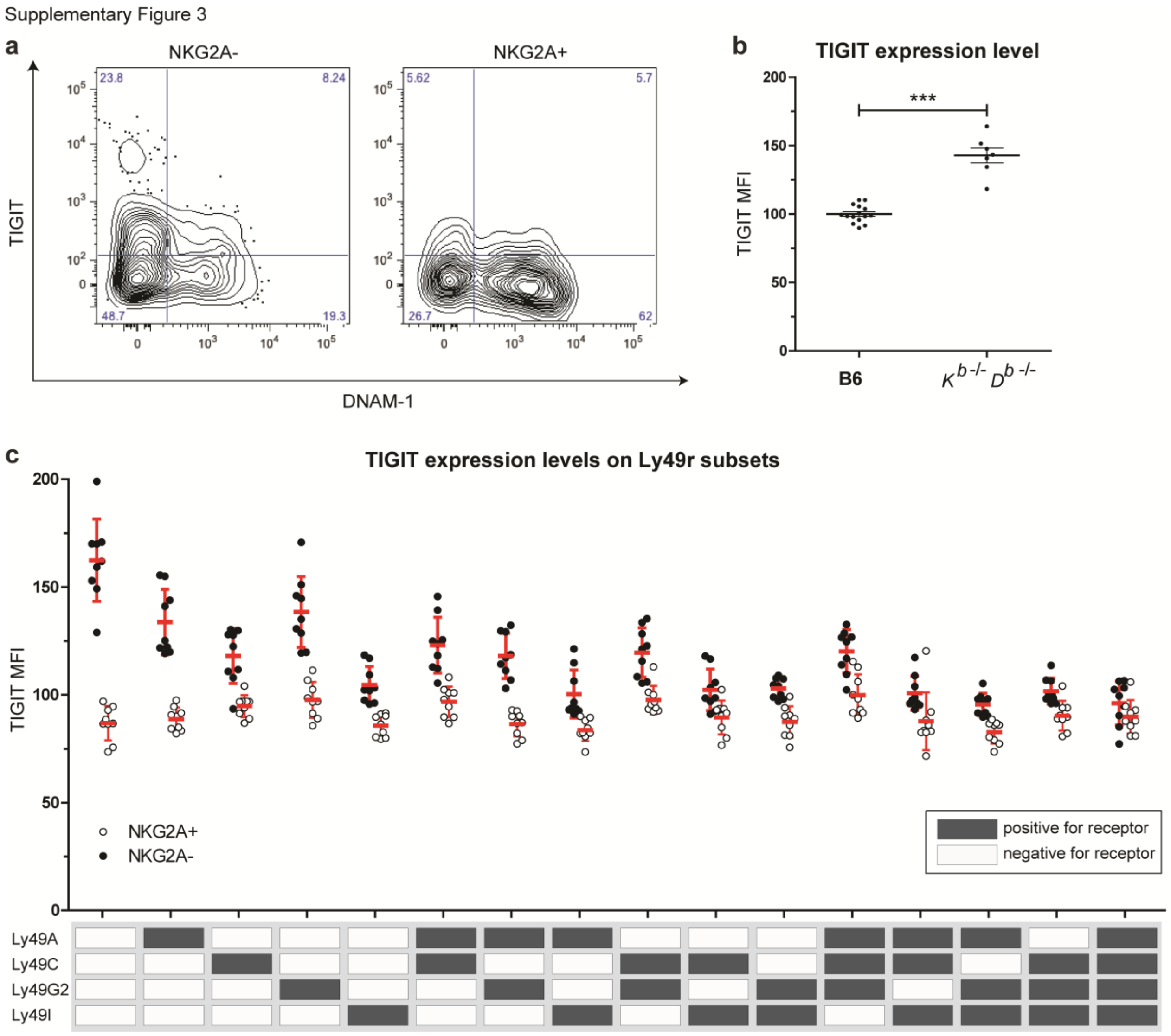

Supplementary Figure 3 TIGIT expression on NK cells and education state. (a) Gating on live NK cells, NKG2A$^-$ NK cells express more TIGIT than NKG2A$^+$ NK cells, one representative plot shown **(b)** Expression levels of TIGT on NK cells from B6 (n=15) and $K^{b-/-}D^{b-/-}$ mice (n=7) **(c)** Expression level of TIGIT on NK cell subsets from B6 mice that express a certain combination of inhibitory Ly49r in the presence (white dots) or absence (black dots) of NKG2A (n=9, 3 experiments). Error bars denote standard deviation (SD). P values are depicted as: ***p<0.001.

Supplementary Figure 4

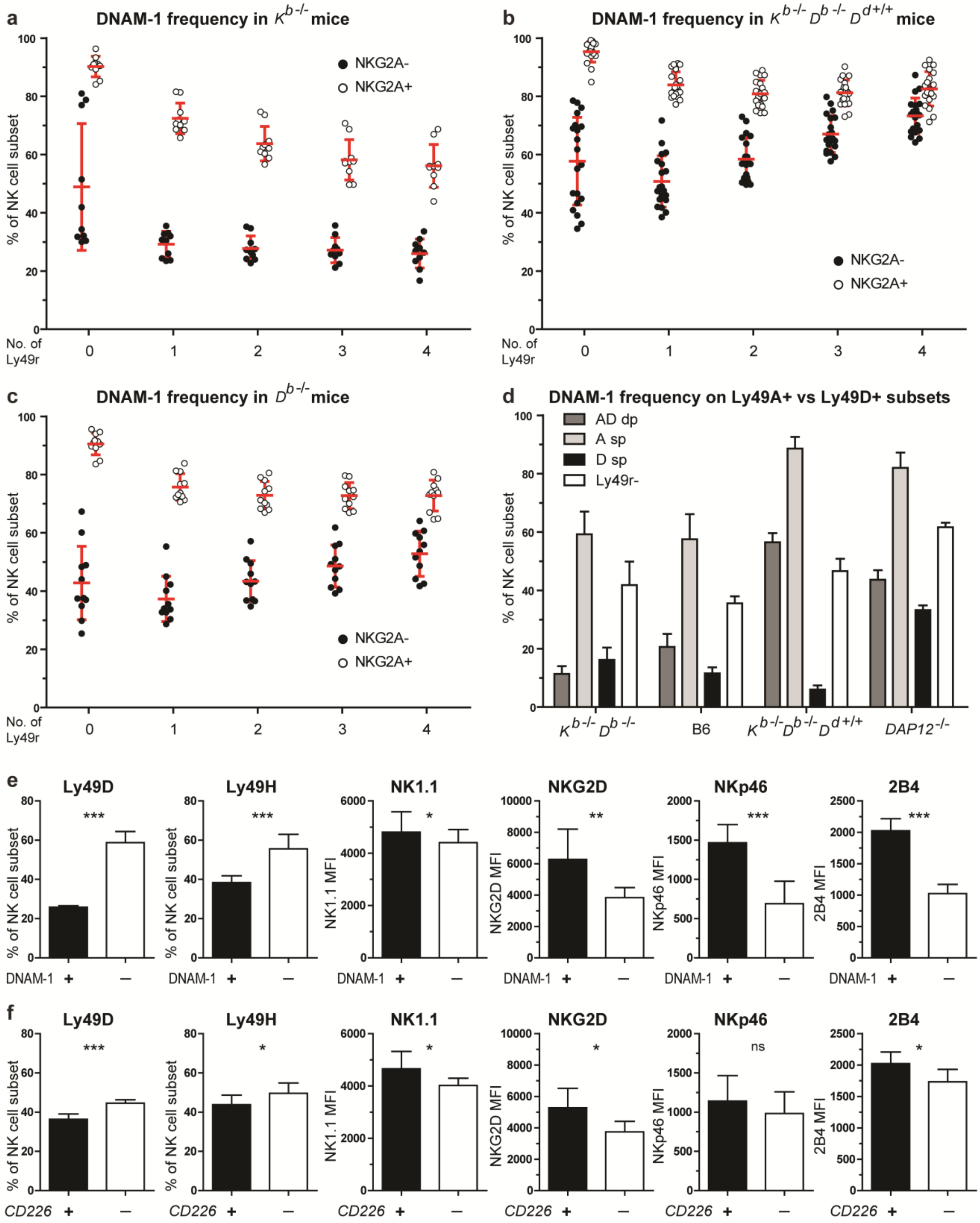

Supplementary Figure 4. Expression of DNAM-1 correlates with the number of Ly49r. (a-c) The number of inhibitory Ly49r on NK cell subsets has an impact on the proportion of DNAM-1^{+} cells within that subset. NK cells of $K^{b-/-}$, MHC-I-deficient D^{d}-transgenic mice ($K^{b-/-}D^{b-/-}D^{d+/+}$) or $D^{b-/-}$ mice have been stained for inhibitory receptors as for Fig 2a, and then grouped based on the number of inhibitory Ly49r they express as for Fig. 3. The frequency of DNAM-1^{+} NK cells within each group is shown for NKG2A^{-} (black dots) cells NKG2A^{+} (white dots) NK cells. **(d)**, NK cells of B6, $K^{b-/-}D^{b-/-}$, MHC-I-deficient D^{d}-transgenic mice ($K^{b-/-}D^{b-/-}D^{d+/+}$) or DAP12$^{-/-}$ mice were stained for inhibitory receptors (Ly49A, Ly49C, Ly49G2, Ly49I and NKG2A), DNAM-1 and Ly49D. Shown is the proportion of DNAM-1^{+} cells of the NK cell subsets that express either Ly49A (A SP), Ly49D (D SP), both of these Ly49r (AD DP) or no Ly49r (Ly49r-). **(e-f)** Expression of activating receptors Ly49D, Ly49H, NK1.1, NKG2D, NKp46 and 2B4 on DNAM-1^{+} versus DNAM-1^{-} NK cells in B6 mice **(e)**, or on NK cells from B6 mice (*CD226*$^{+}$) versus *CD226*$^{-/-}$ mice. Error bars denote standard deviation (SD). P values are depicted as: *p<0.05, **p<0.01, ***p<0.001.

Supplementary Figure 5

a **Coefficientplot of the 32 NK cell subsets for PC 1**

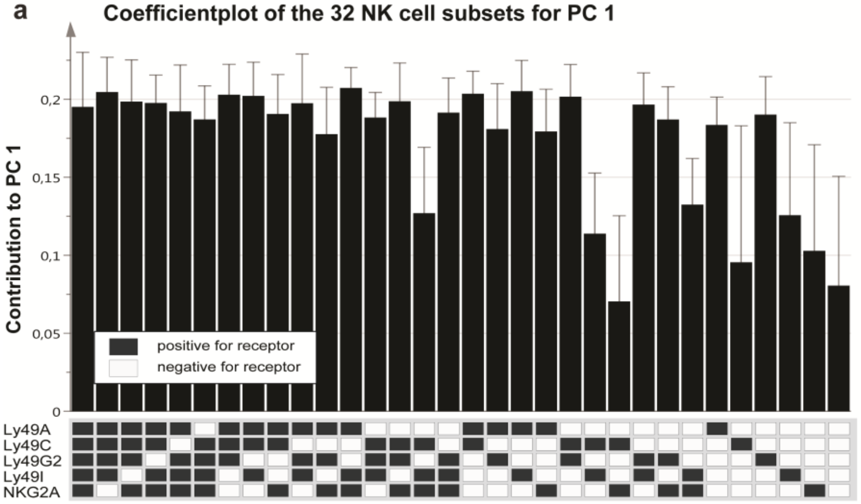

b **OPLS-DA of DNAM-1 frequency on 16 NK cell subsets**

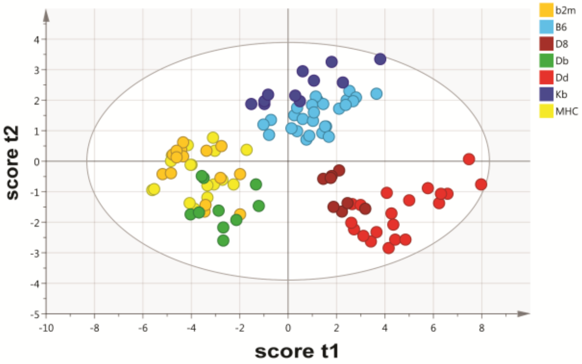

c **Coefficient plots of OPLS-DA for 6 mouse strains reveals relative contribution of NK cell subsets**

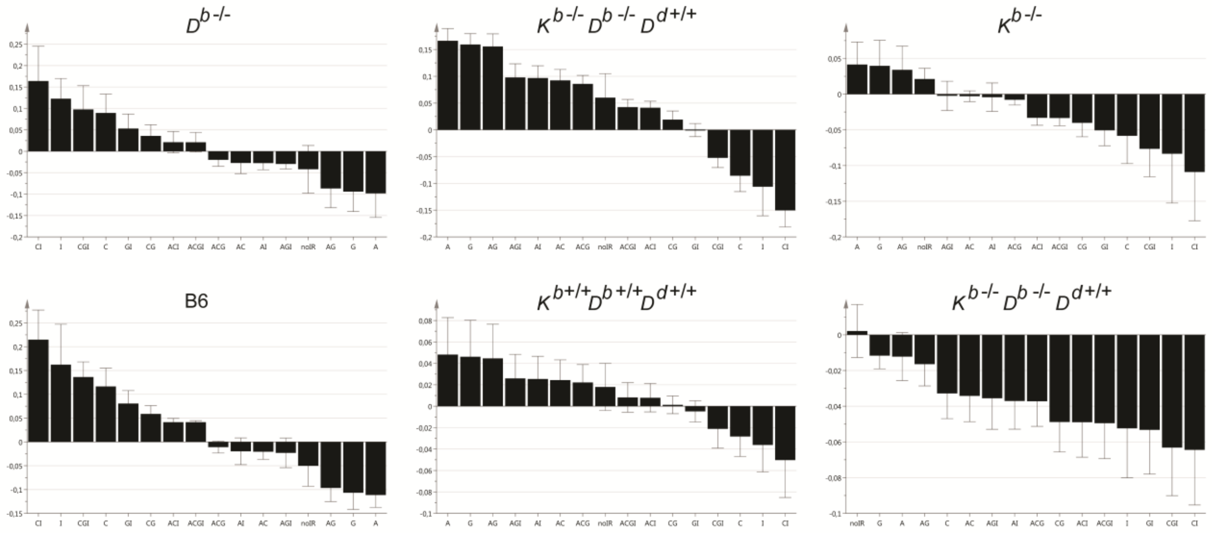

Supplementary Figure 5. Multiple regression analysis of DNAM-1 frequency on Ly49r^{+}NKG2A^{-} subsets. (a) Principal component analysis (PCA) on B6, $K^{b-/-}D^{b-/-}$, $B2m^{-/-}$, $D^{b-/-}$, $K^{b-/-}$, MHC-I-deficient D^{d}-transgenic mice ($K^{b-/-}D^{b-/-}D^{d+/+}$) or D^{d}-transgenic ($K^{b+/+}D^{b+/+}D^{d+/+}$) mice based on the proportion of DNAM-1^{+} cells (DNAM-1 frequency) on the 32 NK cell subsets defined by inhibitory Ly49r and NKG2A expression. NK cells are gated on live singlets NK1.1^{+}CD3^{-} cells. Data are pooled from 8 independent experiments with at least 8 mice per group. Variables contributing to the separation in predictive PC 1 for PCA. Error bars represent the 95 % confidence interval. **(b)** OPLSDA, a supervised multiple regression analysis, shows the difference between pre-defined groups. OPLS-DA was based on the proportion of DNAM-1^{+} cells (DNAM-1 frequency) on the 16 NKG2A-negative NK cell subsets defined by inhibitory Ly49r expression. The model had 2 significant components. Data was pooled from 8 independent experiments. Note that in OPLSDA, groups that overlap cannot be significantly separated from each other. Thus, K^{b}-positive groups, for example, are not significantly different in this system (unless they start to differ in later predictive components). **(c)** Variables characterizing the various mice strains for OPLSDA in Suppl. Fig. 4B. Error bars represent the 95 % confidence interval.

Supplementary Figure 6

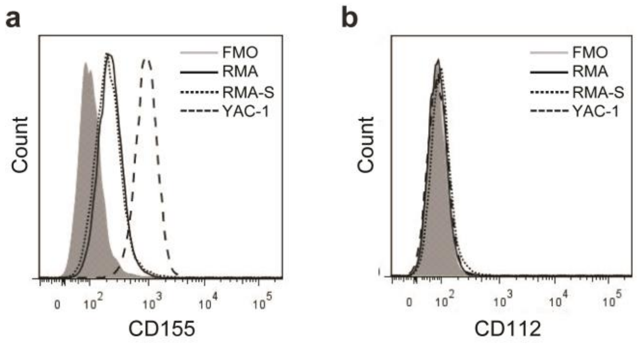

Supplementary Figure 6. RMA and RMAS **have similar levels of DNAM-1 ligands.** *In vivo* propagated RMA and RMAS lymphoma cells and YAC-1 cells were stained for the DNAM-1 ligands CD155 **(a)** and CD112 **(b)**.

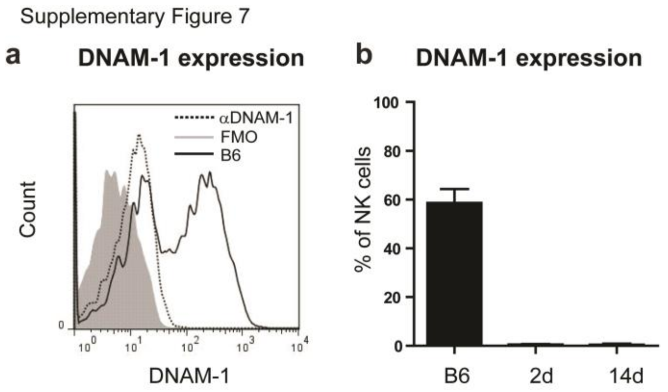

Supplementary Figure 7. ***In vivo* blockade of DNAM-1. (a-b)** Frequency of NK cells in the spleen after 2 days or 2 weeks of DNAM-1 *in vivo* blockade. DNAM-1 was blocked by injection of 200µg of 3B3 (anti-DNAM-1) for 2 days or for 2 weeks every 5 days. Treatment with 3B3 *in vivo* leads to complete blockade of DNAM-1 binding as assessed with clone 10E5. **(a)** Frequency of DNAM-1$^+$ NK cells in the spleen after 2 days or 2 weeks of DNAM-1 *in vivo* blockade. **(b)** NK cell populations summarize data from 2 independent experiments with at least 4 mice per group.

Supplementary Figure 8

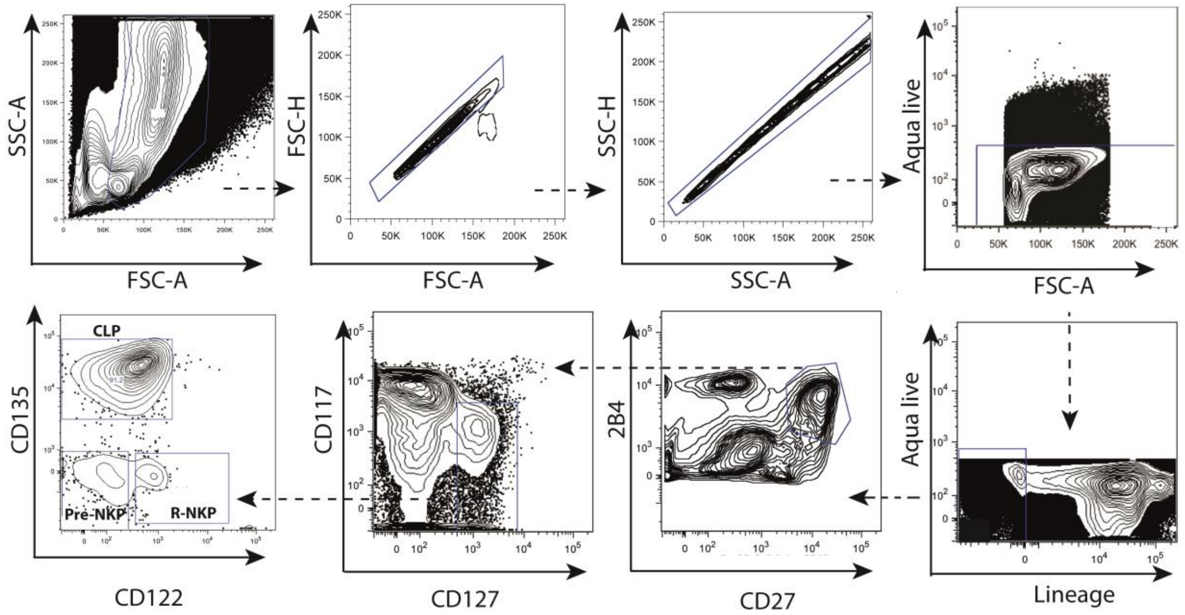

Supplementary Figure 8. Gating strategy for Fig. 7. CLPs, preNKPs, rNKPs and mature NK cells. Gating strategy for CLP, preNKP, and rNKP was described before. Briefly, lineage positive cells ($CD3^+$, $CD19^+$, Gr-1^+, $Ter119^+$, $CD11b^+$, $CD11c^+$, NK1.1^+) were gated out. Then, cells were gated on $CD27^+2B4^+IL7R\alpha^+CD117^-$ population. Subsets were subdivided to CLP ($flk2^+CD122^-$), preNKP ($flk2^-CD122^-$) and rNKP ($flk2^-CD122^+$) subsets

Supplementary Figure 9

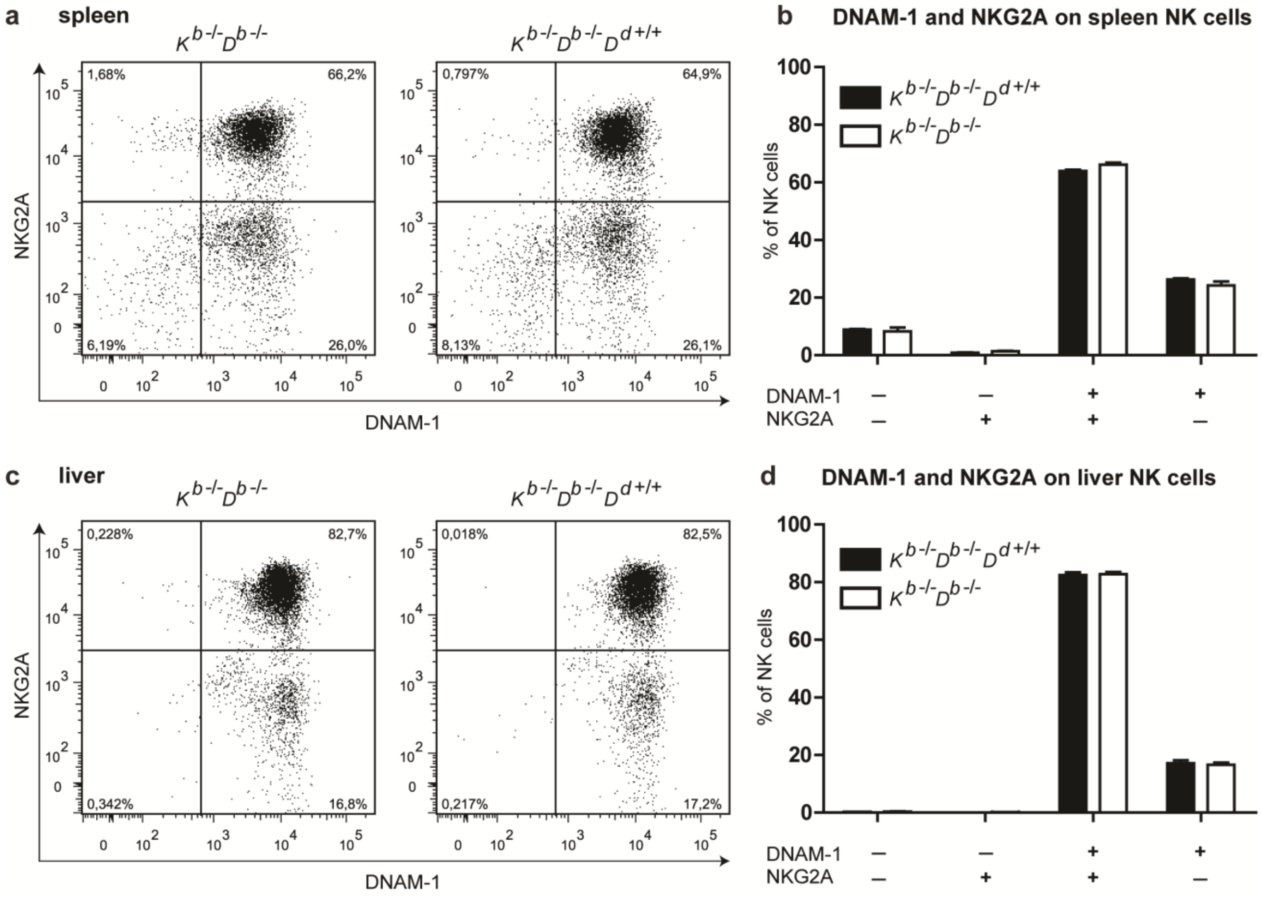

Supplementary Figure 9. DNAM-1 expression during early life. The majority of NK cells from 10 days old mice express DNAM-1 in both spleen **(a-b)** and liver **(c-d)**, regardless of MHC-I expression. **(a+c)** show representative dot plots and **(b+d)** the compiled results of 6 mice from 3 independent experiments. Error bars denote standard deviation (SD).

Supplementary Figure 10

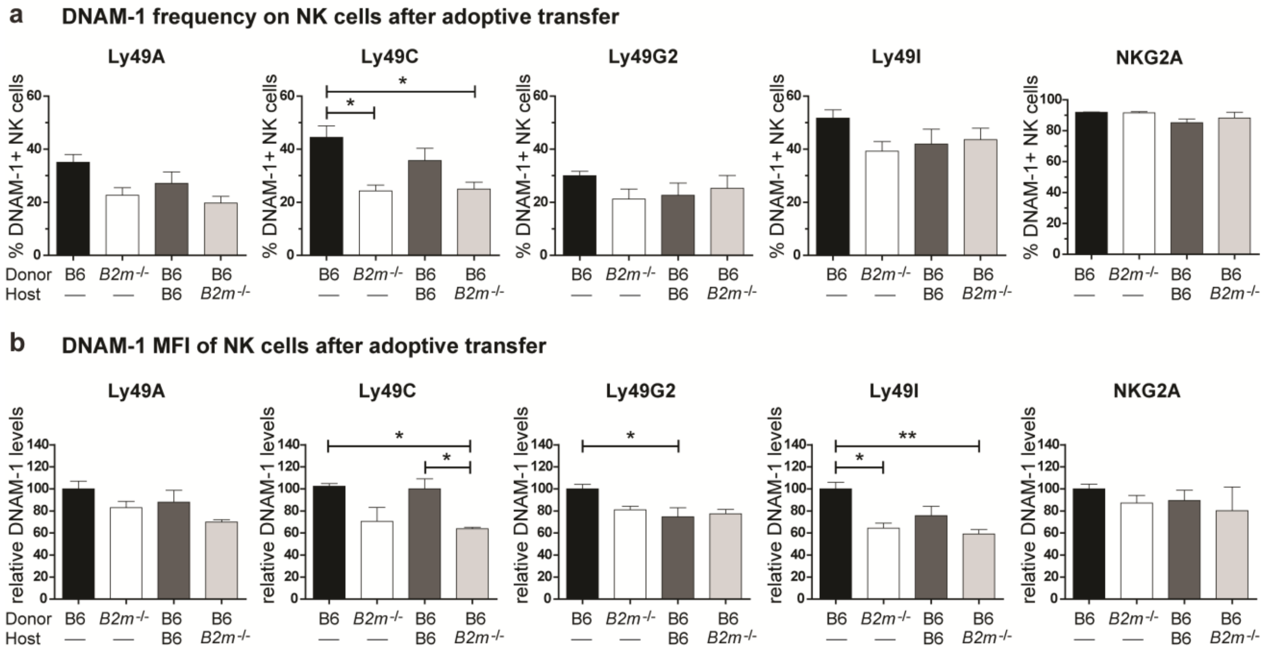

Supplementary Figure 10. DNAM-1 expression is plastic and changes after transfer to an MHCI-different environment. The frequency of DNAM-1 changes after adoptive transfer from B6 to *B2m*$^{-/-}$ mice in the educated, but not the uneducated NK cell subsets. DNAM-1 frequency and DNAM-1 MFI on NK cells after transfer from B6 to *B2m*$^{-/-}$ mice. NK cells from transferred mice are gated on CD45.1^{+}NK1.1^{+}CD3^{-} cells, and SP for the shown inhibitory Ly49r or NKG2A. Expression levels (MFI) are compared to WT B6 controls. Error bars denote standard deviation (SD). P values are depicted as: *p<0.05, **p<0.01.
